# Effects of Dietary Rapeseed and Camelina Seed Cakes on Physical–Technological Properties of Goose Meat

**DOI:** 10.3390/ani12050632

**Published:** 2022-03-02

**Authors:** Violeta Razmaitė, Artūras Šiukščius, Giedrius Šarauskas

**Affiliations:** Animal Science Institute, Lithuanian University of Health Sciences, R. Žebenkos 12, LT-82317 Baisogala, Lithuania; arturas.siukscius@lsmuni.lt (A.Š.); giedrius.sarauskas@lsmuni.lt (G.Š.)

**Keywords:** goose, meat, quality properties, colour, moisture, texture, local breed

## Abstract

**Simple Summary:**

The diets fed to geese traditionally contain more forages and less concentrates, but there are many reasons, including veterinarian and environmental restrictions when intensive rearing should be adopted. It is also important to use different cheaper feed resources for geese, and one of these could be by-products of oil production such as rapeseed and camelina cakes. The present study was aimed at assessing the effects of rapeseed cake and camelina seed cake supplements on the physical and technological attributes of goose meat quality. The oil seed cake-supplemented diets did not reveal any differences in goose meat colour; however, it demonstrated the effects on moisture loss and on texture parameters determined by the texture profile analysis.

**Abstract:**

The objective of the present study was to investigate the effects of commercial diet supplemented with rapeseed and camelina seed cakes on the physical and technological attributes of goose meat quality. The breast and thigh muscles from thirty geese of both sexes of the Lithuanian native breed Vištinės fed the diet containing either rapeseed cake (group 1) or camelina cake (group 2) at the age of 13 weeks were used for the evaluation of physical and technological attributes. The diet did not affect the colour of goose meat; however, females showed higher (*p* < 0.05) values of breast yellowness (b*) and hue angle (h). The camelina group demonstrated higher (*p* < 0.001) cooking losses of breast and thigh muscles and also higher (*p* < 0.05) EZ drip loss and thawing loss of thigh muscles compared with the rapeseed group. Females had higher (*p* < 0.05) cooking loss of the breast, whereas males had higher (*p* < 0.05) cooking loss of the thigh. The growth rate of geese and their slaughter time showed an effect (*p* < 0.05 and *p* < 0.01, respectively) on pH of thigh muscles. Higher (*p* < 0.01) hardness of the breast muscle in the camelina group compared with the rapeseed group was detected by the texture profile analysis (TPA) as well as other parameters such as cohesiveness and gumminess, chewiness. Despite some differences in technological meat quality attributes, the quality of goose meat produced with diet supplementation of 10% of rapeseed cake and camelina seed cake can be considered as suitable.

## 1. Introduction

Global demand of animal products continues to grow. Despite that the consumption of meat has a plateau and pork remains the main meat consumed in Europe, consumers are moving away from beef and sheep meat (which have both fallen more than 40% in Europe between 1970 and 2013) and mainly towards poultry meat, which has grown by 60% over the same period [1]. Although most of the poultry is chicken, other poultry, such as goose meat, also has a consumer because goose meat has a unique flavour and a delicious taste, and people like it also for goose meat’s high nutritional value in terms of optimal composition of essential amino acids as well as its favourable composition of fatty acids, with a high percentage of polyunsaturated fatty acids [2]. Geese raising in Europe is considered relatively popular; however, it does not exceed 0.03% of live poultry production [3] and, therefore, often focuses on seasonal demand [2,4]. Meat quality is an essential issue for consumers. The price of poultry mainly depends on the commercial properties of carcasses. Consumers are more interested in sensorial and nutritional properties, while the meat processing industry gives more attention to the technological properties of meat [5]. Appearance (colour), texture (tenderness, juiciness) and flavour of animal-source foods are crucial attributes to consumers and affect the purchase decisions. Therefore, sensory and technological attributes are considered as core attributes of meat quality [1]. The physical traits of products are relevant to the quality of fresh meat. For consumers, the colour of meat, its water-holding capacity and tenderness play important roles. These traits also can determine the suitability of raw meat for further processing and influence the economic aspect of meat production [1,5]. Moreover, the quality of meat from broiler geese depends on the genotype, age of birds, and their management system, particularly the diet [6,7]. Geese as herbivorous poultry can utilize cheap feed resources and be reared in different systems [2,8,9,10]; however, concentrated ad libitum feeding is widely used [11]. Recently, results have been published regarding the effects of geese feeding with corn and sorghum [12], sugar beet pulp [13], oats and rye [14,15,16,17], soybean meal [18], lupin [6,7]. Considering geese adaptability to roughage, less expensive crop by-products and oil crop by-products such as cottonseed meal [19,20,21] and rapeseed meal and cake [22,23] were used in goose feed. However, the results of the use of different oilseed cakes for goose feeding are scarce. Fu et al. [22] have found that geese had a good adaptation to the diet containing 16% dietary rapeseed meal. Small [24] described that migrating Canada geese frequently die after ingesting large amounts of dry soybeans during the harvest season, because the seeds expand greatly in the moist environment of the digestive tract, causing lethal impaction. Rarely, the same phenomenon has been recorded for other migrating water birds, and for another legume species, such as cowpea but it is not known whether this occurs for Camelina.

The objective of the present study was to investigate the effects of supplementing commercial diets with rapeseed and camelina cakes on goose meat physical and technological properties.

## 2. Materials and Methods

The experiment was conducted according to the National decree-law for the protection of animals used for scientific purposes, harmonized to the relevant European Union directives, and this study was approved by the review board of the Animal Science Institute of the Lithuanian University of Health Sciences (protocol No. 21/03/31/02).

### 2.1. Experimental Material, Design and Diets

The meat of thirty geese of the Lithuanian native breed Vištinės receiving feed with rapeseed cake (group 1) and camelina cake (group 2) from 4 to 13 weeks of age were used for the physical and technological goose meat evaluation. The research material for meat quality evaluation was obtained after chilling of carcasses for 24 h. As the sexual dimorphism of goose is not well-expressed, the sex of every goose (14 females and 16 males) was finally determined by checking the presence of testes or ovarian in carcass.

All geese were fed ad libitum for the entire growing period. For the first 2 weeks, hatched-out unsexed goslings were kept in the room with 22 °C and additionally heated with infrared lamps. Then, the goslings were displaced into the goosery and heated additionally till 4 weeks of age as the temperature was only 17–18 °C in the goosery. Later, the goslings were reared under the natural conditions of light and environment temperature, which was very high in July (25–30 °C); air humidity was 90%. The goslings were hatched out when the natural lighting was L16.5 h: D7.5 h, and during the Southern Hemisphere reached L17.5 h: D6.5 h, and were slaughtered before the natural lighting decreased to L15.5 h: D8.5 h. All the unsexed goslings were kept indoors in concrete pens with sawdust bedding. Each bird was provided with 0.38 m^2^ floor space. The first 3 weeks all goslings received a balanced commercial concentrate feed containing 21% of crude protein, 4% of fat, 3% of fibre, 0.9% of Ca, 0.64% of P, 1.17% of lysine sulphate and 0.54% DL-methionine. From 4 weeks of age up to slaughter, feeding with either rapeseed cake or camelina cake addition was adopted. The composition of feeds and their nutritive value are presented in Table 1.

The goslings were marked with leg rings at the age of 4 weeks, each bird was weighed individually and daily gain from 4 weeks up to slaughter was estimated.

The slaughtering was represented by 3 replications with 5 geese from each group every 4 days. The slaughtering procedures were done using mechanical stunning and by a ventral cut of neck blood vessels within 10 s after the stun and hanged for bleeding. When the birds were considered dead (flaccidity of the body was indicated by a relaxed neck and drooping wings), they were scalded and plucked. The carcasses were eviscerated at approximately 20 min post mortem. The eviscerated carcasses were chilled and stored at +4 °C until meat evaluation on the next day. 

### 2.2. Physical Analysis

#### 2.2.1. pH Measurements

pH was estimated 24 h after slaughter using a digital portable FiveGo^TM^ pH meter F2 (Mettler Toledo GmbH, Schwerzenbach, Switzerland) equipped with a Mettler Toledo puncture-type pH electrode LoT406-M6-DXK-S7/25 with a Xerolyt polymer electrolyte and puncture knife after electrode calibration, as described in the operating instructions of the meter.

#### 2.2.2. Colour Measurements

The five colour parameters of goose breast and thigh muscles were measured using a chromameter CR-410 (Konica Minolta, Osaka, Japan). Measurement and specification of the chromameter were described in our previous study [25]. 

#### 2.2.3. Moisture Losses

The moisture loss was measured in three ways: EZ drip loss, thawing loss and cooking loss. The drip loss was assessed according to the EZ-DripLoss method [26]. To determine the thawing and cooking losses, each sample was weighed before packaging, frozen and stored in the freezer at −65 °C. After being frozen for six weeks, the samples were defrosted. After thawing at 4 °C for 24 h, the muscles were removed from the packaging, blotted with paper towel, and weighed. Then, the samples were cooked and evaluated as described in the previous study [25]. 

#### 2.2.4. Instrumental Evaluation of Texture (It Is Really Instrumental, Not Sensory Evaluation)

The texture of goose breast and thigh muscles was instrumentally measured by Warner–Bratzler shear test (WBSF) and by the texture profile analysis (TPA) on the cooking loss samples using a Texture Analyser TA 1 (Measurement and Calibration Technologies Ametek Comp., Lloyd instruments, Largo, FL, USA) as described in the previous study [25].

### 2.3. Statistical Analysis

The data were subjected to the analysis of variance in the general linear (GLM) procedure in IBM SPSS Statistics 27 with LSD tests to determine the significance of differences of means between the groups. The GLM model included fixed factors of feeding group (rapeseed or camelina cake addition), sex and factor interactions (feeding group × sex). The daily gain of geese and slaughter age/day were included as covariates. The differences were regarded as significant when *p* < 0.05.

## 3. Results and Discussion

### 3.1. Characteristics of Geese Used for Meat Quality Evaluation

An average feed consumption per goose was 17.46 and 16.88 kg in the rapeseed and camelina groups, respectively. No morbidity or mortality of birds occurred in both groups during the test. There were no significant differences in live weight, age and daily gain of slaughtered geese between the rapeseed and camelina groups (Table 2). No significant differences were also noted for these traits between the sexes.

Some authors [27,28] have reported that the age of geese showed an effect on the body and carcass weight of different geese, but in the present study in contrast to other studies, all geese were slaughtered within a week, therefore, the difference in age was negligible and indicated separate slaughter processes rather than the age differences. The body and carcass weights and age were affected by the growth rate (*p* < 0.001). The slaughter day showed an effect (*p* < 0.001) on daily gain. Although the differences in the age between slaughter replications were inconsiderable, during that short time between the slaughter days the birds were growing, increasing daily gain and body weight.

### 3.2. Physical Characteristics of Muscles

Some authors [5,10] reviewed that the colour of poultry meat can be affected by feed, but in the present study there were no significant differences in colour parameters between the feeding groups with the addition of 10% rapeseed or camelina cake. Neither the growth intensity, nor the day of slaughter, had any effect on either the colour parameters of the breast or the thigh (Table 3). However, females showed higher (*p* < 0.05) values of breast yellowness (b*) and hue angle (h) of thigh muscles compared with the males, and this is in agreement with the authors [16,29], who have determined the sex effect on the colour of goose meat. Baéza et al. [5] described the effect of preslaughter conditions on poultry meat pH in their review. Moreover, other authors who studied different goose feeding [6] explained the differences in pH of breast by slaughter process.

In the present study, the camelina group demonstrated lower and higher (*p* < 0.001), respectively, cooking losses of the breast and thigh compared with the rapeseed group (Table 4). Moreover, the camelina group also showed higher (*p* < 0.05) EZ drip loss and thawing loss of the thigh than the rapeseed group. Females had higher (*p* < 0.05) cooking loss of the breast, whereas males had higher (*p* < 0.05) cooking loss of the thigh. The growth rate of geese and their slaughter time showed the effect (*p* < 0.05 and *p* < 0.01, respectively) on the pH of thigh muscles. Juiciness and tenderness of meat are considered to be the most important quality attributes of both fresh meat and meat products. The loss of moisture through drip and cooking losses decreases meat juiciness. Many authors have the opinion that meat juiciness is mainly influenced by the age and genotype and poorly affected by the diet [5]. Goose diets can vary greatly; therefore, there are reports about the diet effect on the drip loss of goose meat [18], but other authors have reported that different feeding did not influence the drip loss [6] or cooking loss [30]. The results obtained in the present study are in agreement with those authors who have found the effect of diet [18] on drip loss of goose meat.

### 3.3. Texture

By sensory evaluation, goose meat was proved to be similar to ostrich meat in the low tenderness aspect [31]; however, many authors who evaluated goose meat texture with the Warner–Bratzler shear force test [32,33,34,35] found diet [30,33], sex and age effects [34]. However, in the present study, no significant differences were observed in goose meat toughness and shear force by the Warner–Bratzler test between rapeseed- and camelina seed-supplemented groups as well as between different sexes (Table 5).

Higher (*p* < 0.01) hardness of the breast muscle was detected by the texture profile analysis (TPA; Table 6) in the camelina group, i.e., in the group with higher moisture loss compared with the rapeseed group. The authors who evaluated the texture of goose meat using the TPA test described associations between goose meat tenderness and meat processing temperature [36]. Other TPA parameters such as cohesiveness and gumminess, chewiness of the breast were also affected (*p* < 0.001 and *p* < 0.05, respectively) by the feeding. There were no significant interactions between the diet group and sex for any tested attribute of goose meat.

## 4. Conclusions

The results of this study revealed that a 10% supplement of rapeseed cake or camelina seed cake did not show any significant differences in goose meat colour; however, camelina seed cake influenced higher moisture loss through cooking losses in the breast and thigh and through EZ drip loss and thawing loss in the thigh. Geese receiving camelina seed cake also demonstrated higher hardness of the breast compared with rapeseed group. The sex appeared to affect meat yellowness (b*) and hue angle (h) of the breast. There were no interactions between the diet group and goose sex. The growth rate and slaughter time showed the effect on the pH of thigh muscles. On the basis of the results obtained for the physical characteristics of goose meat, it can be concluded that goose diet can be supplemented with rapeseed and camelina cakes. The obtained information provides new insights for research into goose production. Further additional investigations of the effects of oil corn by-products on goose meat quality are needed.

## Figures and Tables

**Table 1:** Composition of goose feed and its nutritive value.

Ingredients, %	Group	
	1	2
Wheat	31.12	32.42
Barley	14	14
Maize	10	10
Peas	15	15
Sunflower meal	11.75	10.39
Sunflower oil	1	1
Rape cake	10	-
Camelina cake	-	10
Brewers’ yeast	2.4	2.4
Oyster shell	0.5	0.5
Fodder chalk	1.75	1.79
Premix “Calvet”	1	1
Fodder salt	0.1	0.1
Monocalcium phosphate	1.38	1.4
Calculated nutritional value of concentrates		
Metabolizable energy (ME), MJ/kg	10.79	10.90
Crude protein, g/kg	180.06	180.05
Lysine, g/kg	8.44	8.28
Methionine + cistine, g/kg	6.05	5.87
Fibre, g/kg	58.62	60.69
Ca, g/kg	14.72	14.74
P, g/kg	8.17	8.13
Fat, g/kg	37.67	38.32

**Table 2:** Characteristics of geese used for meat quality evaluation.

Variables	Group		SED	Sex		SED	*p*-Value			
	R *n* = 15	C *n* = 15		F *n* = 14	M *n* = 16		G	S	GR	D
Body weight, kg	4.58	4.45	0.101	4.46	4.56	0.102	0.228	0.339	0.000	0.089
Age, days	88.6	89.4	0.834	89.3	88.7	0.842	0.385	0.464	0.001	-
Daily gain, g	37.0	41.5	2.680	38.4	40.1	0.568	0.108	0.568	-	0.001
Carcass weight, kg	2.83	2.68	0.073	2.71	2.80	0.073	0.055	0.229	0.000	0.253

R-rapeseed; C-camelina; F-female; M-male; G-group; S-sex; GR-growth rate; D-slaughter day; SED-standard error of difference; *p*- values of GLM LSD tests for goose growth rate and slaughter day were significantly different at *p* < 0.001.

**Table 3:** Effects of diet and sex on goose meat colour from different anatomical location.

Colour Parameters	Group		SED	Sex		SED	*p*-Value				
	R *n* = 15	C *n* = 15		F *n* = 14	M *n* = 16		G	S	GR	D
Breast										
L*	42.42	41.69	2.563	43.84	40.27	2.585	0.780	0.180	0.204	0.513
a*	18.51	18.23	0.391	18.50	18.24	0.394	0.482	0.507	0.582	0.398
b*	6.14	6.34	0.423	6.81	5.67	0.426	0.648	0.014	0.625	0.173
C	19.53	19.32	0.445	19.74	19.11	0.449	0.647	0.174	0.494	0.289
h	18.20	19.11	1.081	20.12	17.20	1.090	0.406	0.013	0.865	0.188
Thigh										
L*	41.64	42.26	0.794	41.42	42.48	0.801	0.447	0.195	0.082	0.291
a*	18.57	18.40	0.303	18.79	18.18	0.305	0.571	0.058	0.422	0.538
b*	5.93	6.14	0.362	6.18	5.89	0.365	0.571	0.425	0.922	0.309
C	19.51	19.41	0.364	19.79	19.12	0.368	0.800	0.081	0.480	0.434
h	17.64	18.40	0.883	18.16	17.87	0.890	0.398	0.743	0.807	0.316

R-rapeseed; C-camelina; F-female; M-male; G-group; S-sex; GR-growth rate; D-slaughter day; SED-standard error of difference; L*-lightness; a*-redness; b*-yellowness; C-chroma; h-hue angle; *p* values of GLM LSD tests for goose sex were significantly different at *p* < 0.05.

**Table 4:** Effects of diet and sex on goose meat pH and moisture loss.

Variables	Group		SED	Sex		SED	*p*-Value			
	R *n* = 15	C *n* = 15		F *n* = 14	M *n* = 16		G	S	GR	D
Breast										
pH	5.88	5.86	0.033	5.85	5.89	0.034	0.628	0.295	0.596	0.749
Drip loss, %	2.18	1.99	0.436	2.30	1.87	0.440	0.659	0.337	0.510	0.553
Thawing loss, %	3.98	4.13	0.663	4.13	3.99	0.668	0.824	0.836	0.077	0.567
Cooking loss, %	41.47	38.43	0.830	41.00	38.90	0.837	0.001	0.019	0.711	0.491
Thigh											
pH	6.18	6.12	0.069	6.20	6.09	0.070	0.416	0.121	0.036	0.008
Drip loss, %	0.81	1.52	0.292	1.29	1.05	0.294	0.022	0.434	0.135	0.199
Thawing loss, %	2.71	4.62	0.829	3.29	4.04	0.836	0.031	0.378	0.407	0.895
Cooking loss, %	58.53	61.57	0.830	59.00	61.10	0.837	0.001	0.019	0.711	0.491

R-rapeseed; C-camelina; F-female; M-male; G-group; S-sex; GR-growth rate; D-slaughter day; SED-standard error of difference; *p* values of GLM LSD tests for goose group, sex, growth rate and slaughter day were significantly different at *p* < 0.05 and *p* < 0.001.

**Table 5:** Effects of diet and sex on Warner–Bratzler test parameters on goose meat.

Variables	Group		SED	Sex		SED	*p*-Value			
	R *n* = 15	C *n* = 15		F *n* = 14	M *n* = 16		G	S	GR	D
Breast										
Shear of force, N	1.01	1.09	0.050	1.05	1.05	0.051	0.127	0.870	0.291	0.879
Toughness, N	971.42	983.32	7.057	975.98	978.77	7.117	0.105	0.699	0.436	0.168
Thigh										
Shear of force, N	1.40	1.57	0.136	1.45	1.52	0.137	0.213	0.591	0.651	0.256
Toughness, N	969.87	972.75	15.431	983.91	958.71	15.561	0.854	0.118	0.896	0.967

R-rapeseed; C-camelina; F-female; M-male; G-group; S-sex; GR-growth rate; D-slaughter day; SED-standard error of difference; N-Newtons.

**Table 6:** Effects of camelina and rapeseed diet and sex on goose meat texture evaluated by texture profile analysis.

TPA Parameters	Group		SED	Sex		SED	*p*-Value			
	R *n* = 15	C *n* = 15		F *n* = 14	M *n* = 16		G	S	GR	D
Breast										
Cohesiveness	2.02	2.34	0.086	2.16	2.21	0.086	0.001	0.568	0.060	0.506
Gumminess, N	8.27	11.11	1.075	9.95	9.43	1.084	0.014	0.640	0.220	0.496
Hardness N	16.67	26.04	2.260	21.85	20.87	2.642	0.002	0.715	0.085	0.381
Springiness	0.85	0.84	0.007	0.84	0.84	0.007	0.292	0.744	0.316	0.815
Chewiness, N	7.02	9.36	0.938	8.41	7.97	0.946	0.020	0.646	0.277	0.506
Thigh										
Cohesiveness	2.38	2.52	0.131	2.52	2.38	0.135	0.273	0.306	0.726	0.489
Gumminess, N	7.83	6.79	1.561	5.90	8.72	1.618	0.510	0.089	0.901	0.287
Hardness N	17.99	16.62	3.319	14.90	19.72	3.439	0.682	0.169	0.977	0.517
Springiness	0.82	0.83	0.007	0.82	0.83	0.007	0.692	0.067	0.676	0.173
Chewiness, N	6.44	5.63	1.297	4.81	7.26	1.344	0.535	0.076	0.911	0.264

R-rapeseed; C-camelina; F-female; M-male; G-group; S-sex; GR-growth rate; D-slaughter day; SED-standard error of difference; N-Newtons; *p* values of GLM LSD tests for group were significantly different at *p* < 0.05; *p* < 0.01; *p* < 0.001.

## Data Availability

Data are contained within this article.
